# A high-quality genome of the convergent lady beetle, *Hippodamia convergens*

**DOI:** 10.1093/g3journal/jkae083

**Published:** 2024-04-15

**Authors:** Gavrila Ang, Andrew Zhang, John Obrycki, Arun Sethuraman

**Affiliations:** Department of Biology, San Diego State University, San Diego, CA 92182, USA; Department of Biology, Indiana University, Bloomington, IN 47408, USA; National Institutes of Health, Bethesda, MD 20892, USA; Department of Biological Sciences, California State University San Marcos, San Marcos, CA 92096, USA; Department of Entomology, University of Kentucky, Lexington, KY 40506, USA; Department of Biology, San Diego State University, San Diego, CA 92182, USA

**Keywords:** genomics, lady beetles, phylogenomics, bioinformatics, assembly, annotation

## Abstract

Here, we describe a high-quality genome assembly and annotation of the convergent lady beetle, *Hippodamia convergens* (Coleoptera: Coccinellidae). The highest quality unmasked genome comprises 619 megabases (Mb) of chromosomal DNA, organized into 899 contigs, with a contig N50 score of 89 Mbps. The genome was assessed to be 96% complete (BUSCO). Reconstruction of a whole-genome phylogeny resolved *H. convergens* as sister to the Harlequin lady beetle, *Harmonia axyridis*, and nested within a clade of several known agricultural pests.

## Introduction

In 1842, French entomologist Guerin–Meneville identified *Hippodamia convergens* (Coleoptera: Coccinellidae), a lady beetle native to and widespread throughout North America (Aristiábal and Arthurs 2018). This beetle has an oval body covered by a bright reddish-orange dorsum marked by black dots, akin to other members of the Coccinellidae family. However, it is made morphologically distinct by the eponymous coloring of its prothorax, which consists of a well-defined white border encasing two white converging lines against a black background; hence its common name as the convergent lady beetle. The diet of *H. convergens* mainly consists of aphids, but as a generalist, it can also sustain itself on insect larvae, mites, psyllids, and even nectar (Santos *et al.* 2017). Aphids and psyllids are common pests of agricultural crops such as wheat, tomato, and even rose cultivars (Flint and Dreistadt 2005; Santos *et al.* 2017), and *H. convergens* has been utilized extensively in augmentative biological control of these pests since the early 20th century. One of the earliest records of biocontrol use by the United States occurred in 1903, when *H. convergens* was imported from California to subdue scale insects in Chile (Altieri and Nicholls 1999). Over the years, these beetles have become one of the most commercially available and widely used biological controls against agricultural pests.

The social behaviors of *H. convergens* make it a commercially favorable biocontrol agent. These beetles were found to be transcriptionally upregulated for muscle function and flight during fall (Nadeau *et al.* 2022). This upregulation enables beetles to migrate long distances and aggregate in sheltered sites for the upcoming winter. Studies have attributed the signaling required for aggregation to pheromones n-Tricosane and 2-isobutyl-3-methoxypyrazine (Wheeler and Cardé 2013, 2014). This clustering behavior enables commercial insectaries to easily capture vast amounts of beetles from natural aggregation sites. As a response to lower temperatures and altered photoperiods Obrycki *et al.* (2018), *H. convergens* enter diapause and subsist using fat stores accumulated from hunting in the spring (Nadeau *et al.* 2022). This explains why aphidophagous behavior is not observed in *H. convergens* beetles captured during the fall, despite their remaining near the release site Davis and Kirkland (1982). In response to the rising temperatures during spring and summer, *H. convergens* exit diapause and enter a mating period. Katsarou *et al.* (2005) observed consumption of aphids at higher temperatures (23^∘^C) nearly doubled in comparison to lower temperatures (14^∘^C). However, increased aphidophagy during warmer temperatures, in the context of biocontrol, is confounded by a corresponding increase in flight dispersal behavior as observed by Davis and Kirkland (1982). As many studies have remarked, the suboptimal efficacy of *H. convergens* as a biocontrol agent can be largely attributed to its strong dispersal behavior (Starks 1975; Flint and Dreistadt 2005; Obrycki *et al.* 2009). Still, measuring the efficacy of *H. convergens* as an alternative method to agricultural pest management is difficult to determine given the beetles’ polyphagous and highly mobile nature (Obrycki and Kring 1998; Bjørnson 2008).

The key to optimizing the efficacy of biocontrol agents is through understanding the molecular underpinnings of their biology and socio-behavior. A reference genome will facilitate research in discovering the genetic mechanisms behind feeding behaviors, reproduction, and habitation of biocontrol agents like *H. convergens*. Through the use of third-generation sequencing technology and bioinformatic tools, here we assemble and annotate a high-quality genome for *H. convergens*, and reconstruct a whole-genome phylogeny of publicly available Coleopteran genomes to resolve the position of Coccinellidae.

## Materials and Methods

Inbred lines of *H. convergens*, collected from the wild in Lawrence, Kansas, USA (pers. comm. Drs. JP Michaud, John Obrycki) were maintained in the California State University San Marcos greenhouse in San Marcos, CA on an *ad libitum* diet of pea aphids (*Acyrthosiphon pisum*), raised on crops of fava beans (*Vicia faba*). Sibling clutches of 20 unsexed adults were then pooled and shipped to Dovetail Genomics (now Cantata Bio, Santa Cruz, CA, USA) for processing and sequencing.

### Sequencing

Genomic DNA was extracted from 20 adult beetles using the Qiagen DNeasy Miniprep kit according to the manufacturer’s protocol and quantified on a Qubit 2.0 Fluorometer (Life Technologies, Carlsbad, CA, USA) from all 20 individuals. PacBio sequence libraries were then generated from pooled whole-genomic DNA of lab-reared *H. convergens* beetles using the SMRTbell Express Template Kit 2.0 (PacBio, Menlo Park, CA, USA). Dovetail Genomics utilized the PacBio RS II sequencing system to generate continuous long reads (CLRs) to 30× coverage from the library. Short read libraries were additionally prepared according to the protocols of Putnam *et al.* (2016) (Chicago) and Lieberman-Aiden *et al.* (2009) (Dovetail HiC) with 350 bp mean fragment size using NEBNext Ultra enzymes and Illumina-compatible adapters. Both libraries were sequenced on an Illumina HiSeq X to a target of 30× coverage.

### Data cleaning and quality control

The long reads and Hi-C reads were preprocessed by Dovetail Genomics for the removal of adapter sequences. PacBio CLRs (HiFi) produced thus were consensus derived and therefore, sequences had a predetermined high-base call accuracy so no further quality control was performed. The Hi-C reads were analysed using FastQC (ver 0.11.9) (Andrews *et al.* 2010) to determine base call accuracy.

### Genome assembly

The de novo genome assembler, wtdbg2 (ver 2.5) Ruan and Li (2020), was utilized to construct a genome assembly from long reads. The assembly, initially in the form of a fuzzy De Bruijn graph, was converted into a FASTA file using the consensus tool, wtpoa-cns (ver 2.5). A self-correction method was performed to improve the assembly quality, using both the long reads and Hi-C reads. First, the long reads were aligned to the consensus assembly using Minimap2 (ver 2.24-r1155-dirty) Li (2018). The resulting binary alignment map (BAM) file was sorted according to the sequence order of the long reads files. Samtools sort (ver 1.16.1) Li *et al.* (2009) was used to rearrange the BAM file such that the sequence alignments occurred with respect to the assembly file, or in “genome order”. The sorted BAM file was converted into a suitable input format for wtpoa-cns using the tool Samtools view (ver 1.16.1). This converted file was then used to polish the initial assembly using wtpoa-cns.

The assembly was polished a second time using Hi-C reads as “pseudo” short reads. The assembly was first indexed using bwa index (ver 0.7.17) Li and Durbin (2009) so that it may be efficiently parsed during sequence alignment. The short reads were aligned to this indexed assembly using bwa-mem (ver 0.7.17) Li and Durbin (2009), resulting in a sequence alignment map (SAM) file in the sequence order of the short reads file. Samtools sort was again used to rearrange the mapping file such that the alignments occurred in genome order. Finally, the map was converted into FASTA format using the consensus tool wtpoa-cns.

A de novo genome assembly was also constructed using Canu (ver 2.2) Koren *et al.* (2017)), an assembler that uses a string-overlap graph-based approach. We used an estimated genome size set to 450 megabases (Mbps), based on the genome sizes of *Harmonia axyridis* Chen *et al.* (2021) and *Coccinella septempunctata* Crowley *et al.* (2021).

An additional third de novo genome assembly was constructed using Flye (ver 2.8.1-b1676) Kolmogorov *et al.* (2019), another assembler based on De Bruijn graphs. This tool also requires an estimation of the genome assembly size, which as before, was set to 450 Mbps. In the final stage of the assembly process, Flye executed a single round of self-polishing using the long reads.

Genome completeness of all assemblies was measured using BUSCO (ver 5.4.2) (ref: endopterygota_odb10) Simão *et al.* (2015), which determines the percentage of highly conserved regions within the assembly, as compared against a database of single-copy orthologous regions. We then picked the most complete assembly for all further steps.

### Masking repeats

The most complete assembly (Canu) was screened and masked for interspersed repeats and low complexity DNA sequences using RepeatMasker (ver 4.1.5), with the RMBlast search engine Smit (2015). RepeatMasker was also used to generate an annotation file for repeat regions found within the assembly.

### Removal of contaminating sequences

We used the NCBI’s FCS-GX tool Astashyn *et al.* (2023) to identify contaminant sequences, which aligns sequences against the “all” database, which contains genomic sequences in the BLAST “nt” database, classifies them by taxonomy, identifies contaminating sequences, and then removing them from the purged assembly.

### Removal of duplicated sequences

The repeat-masked and decontaminated assembly was then input into purge-dups (ver 1.0) (Guan *et al.* 2020) tool for the identification and removal of erroneous duplicate sequences. purge-dups uses read depth information to remove potentially erroneous sequences from the assembly: contigs with low coverage (haplotigs), contigs with extremely high coverage (duplicates), and contig overlaps.

### Genome scaffolding

Next, the purged assembly was scaffolded, using the software tool RagTag scaffold (ver 2.1.0) created by Alonge *et al.* (2022). RagTag leverages reference genomes to rearrange and align draft assemblies to form longer sequences. Gaps between high-confidence adjacent query sequences are filled with “N” characters. The reference genomes of *Harmonia axyridis* and *Coccinella septempunctata* were utilized to create two respective, scaffolded assemblies. It is important to note that RagTag does not modify the input query sequences; it solely reorders, reorients and gap-fills. Further scaffolding was performed using the Hi-C data using the SALSA2 pipeline Ghurye *et al.* (2017)—briefly, paired-end Hi-C reads were mapped to the scaffolded genome using bwa-mem2 v 2.2 Vasimuddin *et al.* (2019), then provided as input to SALSA2 for scaffolding and gap-filling.

### Assembly validation

Finally, the scaffolded assembly was assessed according to the 3C criterion (contiguity, completeness, and correctness). Assembly contiguity was measured using QUAST (ver 5.2.0) (Gurevich *et al.* 2013), which yielded N50, L50 statistics, contig/scaffold sizes, and estimated number of misassemblies. Genome completeness and correctness were measured using BUSCO (ver 5.4.2) (ref: endopterygota_odb10), as described above. Additionally, we assessed k-mer completeness, genome size, and genomewide heterozygosity using jellyfish v.2.3.1 (Marçais and Kingsford 2011) with a kmer length of 19 (estimated as the best kmer size using meryl v.1.3 (Rhie *et al.* 2020)), followed by analyses of the k-mer histograms using GenomeScope (Vurture *et al.* 2017). We additionally assessed k-mer completeness in the final assembled genome using merqury v.1.3 Rhie *et al.* (2020).

### Gene prediction and annotation

RepeatMasker (v. 4.1.5) (Smit *et al.* 2015) was used to produce an annotation of the interspersed and tandem repeat sequences in the assembly against Coleopterans in the CONS-Dfam_3.7 database. It was also used to create a new “soft-masked” assembly, wherein repetitive sequences are in lowercase. This “soft-masked” assembly was input into Augustus (ver 3.5.0) (Stanke *et al.* 2006) for *ab initio* gene prediction, using the pre-trained gene model of *Triboleum castaneum*, as a reference. The gff file produced by Augustus and the “soft-masked” assembly was input into JBrowse (ver 1.16.11) (Buels *et al.* 2016) to create a visual map of the coding sequences within the *H. convergens* genome.

### Phylogenetic analysis

To phylogenetically resolve the position of *H. convergens* in the Coleopteran tree of life, we constructed a BLAST database of all predicted genes from our genome annotation step, and intersected these against single-copy orthologs identified among other Coleopteran genomes and the genome of *Harmonia axyridis* (Chen *et al.* 2021). Multiple sequence alignments of all such 3,865 used protein-coding genes that were represented in all species analyzed were constructed using pasta v.1.8.6 Mirarab *et al.* (2015). Individual gene trees were then constructed using RAxML v. 8.2.12 (Stamatakis 2014) with the PROGRAMMAJTTF mutation model (sensu Thomas *et al.* (2020)). An unrooted species tree was then constructed using all 3,865 gene trees with ASTRAL v.5.7.7 (Zhang *et al.* 2018).

## Results and discussion

A total of 267 gigabases of consensus long reads in the form of 3 FASTA files were derived from the CLRs by Dovetail Genomics from PacBio HiFi reads, while 86 gigabases of Hi-C paired-read data (43 gb of forward and reverse reads) was produced.

The final Canu assembly yielded 8,520 contigs of 1,417 mbps, with a contig N50 score of 0.24 and a BUSCO score of 97.7%, with notably high proportion of orthologs with duplicates of 80.7%, as against single-copy orthologs (Table 1). These duplicated sequences from the de novo assembly were removed to improve assembly correctness, using the purge-dups tool resulting in a drop in overall BUSCO scores of 82.1%. However, the proportion of duplicated orthologs also notably decreased, successfully removing 26.3% of duplicated orthologs. After purging, the length of the final Canu assembly shortened by 48%, falling closer to the expected genome length of Coleopterans (Table 2).

**Table 1.** Comparative assembly quality between the Canu assembly, and subsequent correction steps (purging of duplicates, scaffolding), length, and completeness statistics as assessed against the BUSCO endopterygota_odb10 database.

Statistic/assembly	1	2	3	4	5	6	7	8
Total length (Mb)	1,428	750	2,429	951	1,418	620	620	619
No. of contigs	16,181	4,933	32,016	6,945	8,520	2,174	899	422
Contig N50 (Mb)	0.20	0.45	0.32	0.58	0.24	0.62	0.62	0.62
Scaffold N50 (Mb)	0.20	0.45	0.33	0.58	0.24	0.62	89.67	89.67
Complete BUSCO score (%)	93.20	82.10	97.70	85.40	97.70	95.90	96.10	96
Complete and single-copy BUSCOs (%)	47.70	62.90	17	28.70	10.90	75.60	78.70	78.70
Complete and duplicated BUSCOs (%)	45.50	19.20	80.70	56.70	86.80	20.30	17.40	17.30

Assemblies shown are (1) wtdbg2, (2) wtdbg2+purge_dup, (3) Flye, (4) Flye+purge_dup, (5) Canu, (6) Canu+purge_dup, (7) Canu+purge_dup+RagTag, (8) Canu+purge_dup+RagTag+FCS-GX+salsa2.

**Table 2.** Comparison of assembly correctness, contiguity and completeness between the *Hippodamia convergens* assembly from this study, *Harmonia axyridis*, and *Coccinella septempunctata*.

	3C criterion	*Hippodamia convergens*	*Harmonia axyridis*	*Coccinella septempunctata*
Total length (Mb)		619	426	399
Contig N50 length (Mb)	Contiguity	89.67	22.90	16.50
Overall BUSCO score (%)	Completeness	96.10	97.40	97.50
Duplicated BUSCOs (%)	Correctness	17.40	2.30	1

The final draft assembly (purged of duplicates, decontaminated, and scaffolded), achieved a BUSCO score of 96.0% and decreased the number of contigs by 59% and increased the contig N50 score to 89 Mb, thus improving assembly contiguity. Scaffolding using RagTag also made a modest increase in assembly size by 127 kilobase-pairs due to gap filling with “N” sequences. 11.4% of the genome assembly was determined to contain repeats (Table 3).

**Table 3.** Repeat content variation assessed on the scaffolded Canu assembly of *Hippodamia convergens* using RepeatMasker.

		Number of elements	Length occupied(bp)	% of sequence
Retroelements		140,220	55,733,568	3.84
	SINEs:	1	63	0
	Penelope:	0	0	0
	LINEs:	105,258	35,902,146	2.47
	CRE/SLACS	0	0	0
	L2/CR1/Rex	42,197	10,633,373	0.73
	R1/LOA/Jockey	11,860	4,723,341	0.33
	R2/R4/NeSL	4,708	2,603,344	0.18
	RTE/Bov-B	21,472	10,060,078	0.69
	L1/CIN4	0	0	0
	LTR elements:	34,961	19,831,359	1.37
	BEL/Pao	10,477	5,368,518	0.37
	Ty1/Copia	3,989	2,732,450	0.19
	Gypsy/DIRS1	20,495	11,730,391	0.81
	Retroviral	0	0	0
DNA transposons		175,613	32,941,932	2.27
	hobo-Activator	35,276	3,710,132	0.26
	Tc1-IS630-Pogo	113,343	24,926,017	1.72
	En-Spm	0	0	0
	MULE-MuDR	110	12,308	0
	PiggyBac	492	111,551	0.01
	Tourist/Harbinger	5,365	902,756	0.06
	Other (Mirage, P-element, Transib)	755	119,066	0.01
Rolling-circles		3,937	477,624	0.03
Unclassified:		345,298	60,864,377	4.19
Total interspersed repeats:			149,539,877	10.31
Small RNA:		1,648	734,047	0.05
Satellites:		0	0	0
Simple repeats:		282,696	12,335,559	0.85
Low complexity;		46,945	2,355,942	0.16

The assembly quality was validated based on the 3C criterion of completeness, contiguity, and correctness. Assembly statistics were generated using the tools QUAST and BUSCO. QUAST was employed to measure genome contiguity, with the final *H. convergens* assembly achieving a contig N50 length of 89 Mb. Genome completeness was estimated using the proportion of complete BUSCOs, whilst the proportion of complete duplicated BUSCOs served as a proxy for genome correctness in the absence of a reference genome. The assembly protocol achieved an overall BUSCO score of 96.0%, indicating a high level of completeness (complete:96.0% = single-copy orthologs:78.7% + duplicate:17.3% + fragmented:1.5% + missing:2.5%; n:2,124). However, it was observed that 17% of the BUSCOs were duplicated, indicating room for improvement in genome correctness. Still, the de novo assembly size of *H. convergens* is comparable to other Coleopteran genomes. We also acknowledge that these duplicated BUSCO’s could potentially also arise from inherent heterozygosity and thereby isoforms present amongst the 20 adult beetles that were utilized to generate the assembly. merqury v.1.3 Rhie *et al.* (2020) estimated a haploid genome size of 426.32 Mbps, which still remains 200 Mbps smaller than the estimated best assembly. Similar analyses using GenomeScope v.1.0 estimated a haploid genome size of 383 Mbps, with a genomewide heterozygosity of 0.041.

The assembled genome was analysed using the gene prediction tool AUGUSTUS, with a trained model based on the *Tribolium castaneum* geneset. The resulting gene predictions were then stored in a GFF file. This GFF file, in addition to the transcriptomic data provided by (Nadeau *et al.* 2022), was used to annotate 154,668 genic sequences in the assembly. This genome annotation was visualized in a genome browser generated by JBrowse and made publicly accessible.

**Fig. 1.**
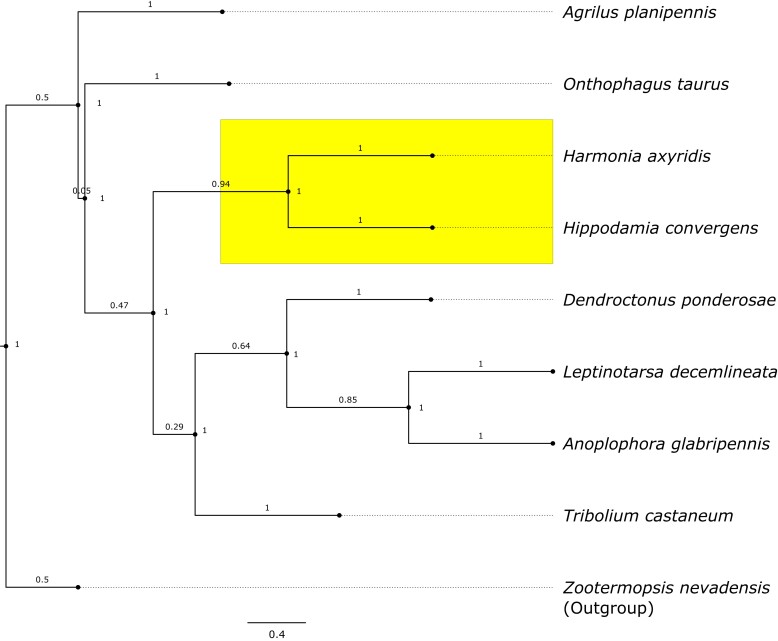
ASTRAL Species tree reconstruction amongst Coleopterans, resolving the omnivorous Coccinellid monophyly, comprising *Hippodamia convergens* and *Harmonia axyridis*, which in turn share a common ancestor with several species of herbivorous agricultural pests. Branch values indicate ASTRAL quartet scores (between 0 and 1), indicating support for the branch, while node values indicate support for the split (1 = 100% of gene trees support the split).

The positioning of *H. convergens* within the Coleopteran order was resolved through 3,865 complete genes conserved across the beetles. The large number of orthologous genes used amongst the beetles provided confidence in the placement of *H. convergens* within the broader Coleopteran phylogeny (Fig. 1). These findings pave the way for future research projects focused on investigating the specific genes associated with predatory and social behaviors Yuan *et al.* (2022). Utilizing a well assembled and annotated genome can help researchers gain a deeper understanding on the invasive potential of *H. convergens*, its inclination towards frugivory, and its potential impact towards native arthropod populations. In particular, population genetic analyses using the assembled *H. convergens* genome can model the risks associated with uncontrolled release of *H. convergens*, including its potential to disrupt local ecosystems. Further research in this direction will contribute to informed decision-making and the design of management strategies to mitigate potential negative consequences associated with *H. convergens* mediated biocontrol.

## Data Availability

All scripts used in assembly, quality control, and annotation are available on the project’s GitHub page: https://github.com/gavrila-ang/MetaGenome The genome assembly has been deposited with NCBI Project Accession: PRJNA1017495. The JBrowse instance of the predicted genome annotation can be accessed at https://usegalaxy.org/datasets/f9cad7b01a4721357d2c38a73df02de6/display/?preview=True.
